# spind: an R Package to Account for Spatial Autocorrelation in the Analysis of Lattice Data

**DOI:** 10.3897/BDJ.6.e20760

**Published:** 2018-02-28

**Authors:** Gudrun Carl, Sam C Levin, Ingolf Kühn

**Affiliations:** 1 Helmholtz Centre for Environmental Research - UFZ, Halle (Saale), Germany; 2 Martin Luther Universität Halle-Wittenberg, Halle (Saale), Germany; 3 German Centre for Integrative Biodiversity (iDiv), Leipzig, Germany

**Keywords:** Cohen's kappa coefficient, Generalised Estimating Equations, Goodness-of-fit, Multimodel Inferrence, Multiresolution Regression, Prediction accuracy, Spatial autocorrelation, Species distribution modelling, Wavelet Revised Models

## Abstract

*spind* is an R package aiming to provide a useful toolkit to account for spatial dependence in the analysis of lattice data. Grid-based data sets in spatial modelling often exhibit spatial dependence, i.e. values sampled at nearby locations are more similar than those sampled further apart. *spind* methods, described here, take this kind of two-dimensional dependence into account and are sensitive to its variation across different spatial scales. Methods presented to account for spatial autocorrelation are based on the two fundamentally different approaches of generalised estimating equations as well as wavelet-revised methods. Both methods are extensions to generalised linear models. *spind* also provides functions for multi-model inference and scaling by wavelet multiresolution regression. Since model evaluation is essential for assessing prediction accuracy in species distribution modelling, spind additionally supplies users with spatial accuracy measures, i.e. measures that are sensitive to the spatial arrangement of the predictions.

## Introduction

Grid-based data sets, whether they are biodiversity data such as species distributions, species abundance or trait data or sets of ecosystem service values, sociological or economic indices, of concentrations of pollutants, soil or hydrological properties, are often used in spatial modelling and may exhibit some degree of spatial dependence. This means that sampling at nearby locations may lead to sample values that are more similar than those further apart (Tobler 1970, Legendre 1993). In such cases, the assumption of independently and identically distributed errors is violated in standard regression models. Consequently, estimates of standard errors and type I error rates can be biased (Dormann et al. 2007, Lennon 2000). Although a variety of spatial statistical methods taking the spatial autocorrelation into account already exist in *R* (R Core Team 2016), there is, to our knowledge, still a lack of user-optimised models running for both presence/absence (binary response) and species abundance data (Poisson or normally distributed response) and, last but not least, running as computationally fast and efficient procedures. It is believed that the spatial statistical methods included in the** spind** package and described here can contribute to filling this gap.

Various spatial methods are available to account for spatial autocorrelation in multiple linear regressions (e.g. Dormann et al. 2007, Beale et al. 2010, Hefley et al. 2017). Here, two methods are offered that are based on the two fundamentally different approaches of generalised estimating equations (GEE) (Zeger and Liang 1986, Yan and Fine 2004, Carl and Kühn 2007) on the one hand and wavelet-revised methods (WRM) (Carl and Kühn 2008, Carl and Kühn 2010) on the other. These methods are extensions of the generalised linear model (GLM). Moreover, **spind** provides multi-model inference functions for both approaches. Additionally, spatial scaling is possible, for instance, with wavelet multiresolution regression (WMRR). WMRR is a scale-specific regression that, when combined with a multimodel inference approach, is able to evaluate the relative importance of several variables across different spatial scales. In addition to these methods and their helper functions, **spind** supplies users with functions for assessing prediction accuracy. In a spatial context, the traditional non-spatial accuracy measures can be misleading, particularly when sampling on raster maps. Therefore, **spind** provides generalised, spatially corrected performance metrics that are sensitive to the spatial arrangement of the predictions and are analogous to classical metrics. Specifically, they take into account the proximity of modelled to observed (actual) values by still being based on and comparable to measures such as AUC, Kappa, sensitivity, specificity or True Skills Statistics.

## Installation

Package **spind **is available on CRAN and can be downloaded there. See: https://CRAN.R-project.org/package=spind. The development version is available on GitHub as well. See: https://github.com/levisc8/spind. 

*# Install from CRAN*

*install.packages('spind')*

*#* *Install development version from GitHub*

*devtools::install_github('levisc8/spind')*

## Usage

### Wavelet-Revised Models (WRM)

Next, the other main model that is introduced in this package is explained- the wavelet-revised model (Carl and Kühn 2010). WRMs, like GLMs, can be used to fit linear models for response variables with different distributions: Gaussian, binomial or Poisson. WRMs, like GEEs, are extensions of GLMs for autocorrelated variables, i.e. models in which the residuals can be autocorrelated. As wavelet-based methods, however, they are mathematically quite different to GEEs. The crucial idea behind wavelet analysis can be formulated as follows: wavelets are small waves, that is, localised oscillating functions. Such a brief oscillation can locally be compared with a segment of a given data set. A corresponding wavelet coefficient is able to capture the degree of similarity. Shifting the wavelet along the data set, this comparison can be done at several locations. Furthermore, one can reanalyse the data set with gradually compressed or stretched wavelets, i.e. wavelets of different oscillating behaviour, which correspond to different scales or resolutions. Viewed from this perspective, each wavelet acts as both *window* and *filter*. The wavelet characterised by a certain, strictly limited range opens a window to a subset of data belonging to this area and the wavelet selecting a frequency to be investigated helps for feature extraction in this area.

This means that wavelet analysis can be used for spatial filtering similar to the principal coordinates of neighbour matrices (PCNM) analysis (e.g. Borcard et al. 2004) or its generalisation, the Moran’s eigenvector maps (MEM) approach (Dray et al. 2006). However wavelet analysis is a refinement of such methods. While PCNM/MEM methods are statistical versions of Fourier analysis (i.e. the spatial eigenvectors that they use for filtering are basically sinusoidal waves spread out over the whole spatial data set), wavelets (i.e. small waves visualisable as localised oscillations) additionally act as windows. Therefore, PCNM/MEM and wavelet methods differ in their ability to detect local variations and to make local adjustments (Carl et al. 2016). Wavelet analysis is locally more accurate compared to Fourier analysis. It has proven to be a suitable method to quantify spatial structure as a function of both location and scale (Carl and Kühn 2008).

The number and kind of coefficients in discrete wavelet transforms depend on the number and kind of wavelets used in the analysis. Briefly, there are two kinds of coefficients: *detail* and *smooth* ones. These reflect the different oscillating behaviour and distinguish between highly varying (detailed) and slowly varying (smooth) parts. This enables the user to decompose and to filter a data set in relation to its locally varying frequency characteristics. The wavelet filters are implemented using wavelet transforms from the **waveslim** package (Whitcher 2015).

Different from other methods such as PCNM/MEM, the wavelet filters in thisapproach are applied to the response variable as well as all explanatory variables in a multiple regression. Moreover, they are applied within every step of GLM iteration before the regression coefficients are computed (Carl and Kühn 2008, Carl and Kühn 2010). This process of pre-filtering within GLM iteration is carried out without any additional covariates or regression coefficients. That is why this approach is also not comparable to a geographically weighted regression. Instead, it is a single GLM performed on consistently filtered variables. 

Spatial autocorrelation occurs when data sampled at adjacent locations exhibit more similar values than distant ones. This feature is detectable by the coefficients of smooth and relatively small wavelets. In WRMs, therefore, such kind of coefficients are always removed and thus smooth (i.e. low frequency) parts of data and, as a consequence, spatial autocorrelation. The tuning parameter *level* is a preset for the window size, that is, the range of all smooth wavelets. Sequentially setting *level* to lower integers allows the method to be adapted to one's data and autocorrelation gradually reduced, where strongest reduction can be found at the finest resolution, with *level=1* usually working best.

For illustration, WRM is presented using the same musdata data set as above.

*mwrm <- WRM(musculus ~ pollution + exposure, family = "poisson", data = musdata,*

*coord = coords, level = 1, plot = TRUE)*

*summary(mwrm)*

*predictions <- predict(mwrm, newdata = musdata)*

All calculations can be performed using different types of wavelet families and different types of wavelet transforms, where *haar* for wavelet and *dwt* for transform is the default. To minimise boundary effects, WRM offers different options for embedding data collected in two-dimensional space in the larger frame of a square matrix. This can be done either by padding with zeros, mean values or reflected values at boundaries acting like mirrors. Therefore, there are different settings for padform in *pad=list(…)*. Moreover, there is a factor *padzone* in *pad=list(…)* for expanding the padding zone.

WRM has many of the same features as GEE. Setting *plot=TRUE* allows the examination of the autocorrelation of residuals from a GLM of the same family as the WRM and passing further *ggplot2* functions to *customize_plot* allows the user to manually add or subtract the required features (Fig. 2). Methods for WRM (*summary.WRM* and *predict.WRM*) allow the user to examine outputs from the model using the same code as might be used for a GLM. However, note that this reports an AIC (and AICc) score, rather than a QIC score as in the GEE.

### Wavelet multiresolution regression (WMRR)

Having filtered all data sets in relation to their frequency characteristics and removed smooth parts with a WRM, one can additionally decompose the detail (i.e. high frequency) parts into scale-specific subcomponents. One is then able to develop a scale-specific regression technique, subsequently known as wavelet multiresolution regression (Carl et al. 2016). Note that this kind of regression does not aim for autocorrelation removal. Instead, it aims at scale-dependent or cross-scale investigations. In detail, this means that a scale-specific wavelet multiresolution regression, *scaleWMRR*, keeping only detail parts of a certain scale level, accounts for fluctuations or spatial variations at a specific spatial resolution. Moreover, note that *scaleWMRR* at *scale=1* (and for *detail=TRUE*) is the same as WRM at *level=1*, as there are further decompositions into lesser objects only for *scale = 2*. Note that a switch is being made to the *carlinadata* data set now.

*data(carlinadata)*

*coords <- carlinadata[ ,4:5]*

*# scale-specific regressions for detail components*

*ms2 <- scaleWMRR(carlina.horrida ~ aridity + land.use,*

*family = "poisson", data = carlinadata, coord = coords, scale = 2, trace = TRUE)*

*ms3 <- scaleWMRR(carlina.horrida ~ aridity + land.use,*

*family = "poisson", data = carlinadata, coord = coords, scale = 3, trace = TRUE)*

*aic<-aic.calc(carlina.horrida ~ aridity + land.use, family = "poisson", data = carlinadata,*

*mu = ms3$fitted)*

*aic*

It is important to mention that it is essential to avoid comparisons of significance tests across scales. This is due to the fact that the sample size in general changes when scale-specific subcomponents are eliminated. However, in order to provide a consistently good quality criterion, there is the possibility to calculate log likelihoods and AIC values and then estimate the relative importance of a variable using the approach of model selection based on multimodel inference (MMI)(see below).

### Generalised estimating equations (GEE)

The generalised estimating equations approach developed by Zeger and Liang (1986) is an extension of generalised linear models, but in which the outcomes are not assumed to be independent. Therefore, GEEs, by contrast to GLMs, allow for autocorrelated residuals. GEEs, in analogy with GLMs, can be used to fit linear models for response variables with different distributions: Gaussian, binomial or Poisson. Mathematically, the variance of the response is replaced by a variance-covariance matrix which takes into account that observations are not independent. Originally, the approach has been developed for analysing longitudinal data. This approach was modified to use GEE models for spatial, two-dimensional data sets sampled in rectangular grids (Carl and Kühn 2007). This package utilises the functions already written for GEEs from the packages **gee** (Carey 2015) and **geepack** (Yan 2002, Yan and Fine 2004, Halekoh et al. 2006). These spatial GEEs are adapted for easy use in the context of spatial modelling and can handle the following correlation structures (corstr argument): 1) independence: this is the same as a GLM, because the identity matrix is used as correlation matrix. 2) fixed: all correlation parameters are fixed and the correlation structure is predetermined by an isotropic power function, which is adapted to the residual autocorrelation of the generalised linear model. It will not change during an iterative procedure. 3) Clustered: correlation parameters are to be estimated. To reduce the number of parameters, the variance-covariance matrix is assumed to be of block diagonal form. There are two options. 3a) exchangeable: specifies that all parameters within blocks must be equal. 3b) quadratic: specifies that certain parameters must be equal, with the result that the strength of correlation is always the same at a certain distance. For these cluster models, different values for cluster size cluster are allowed. 

To illustrate the use of the package, a GEE model is presented using the generated musdata data set included in the package. The following code uses already available datasets and provides *R* examples:

*library(spind)*

*data(musdata)*

*# Fit a GEE and view the output*

*coords <- musdata[ ,4:5]*

*mgee <- GEE(musculus ~ pollution + exposure, family = "poisson",*

*data = musdata, coord = coords, corstr = "fixed", plot = TRUE, scale.fix = FALSE)*

*summary(mgee, printAutoCorPars = TRUE)*

*predictions <- predict(mgee, newdata = musdata)*

**Figure 1.**
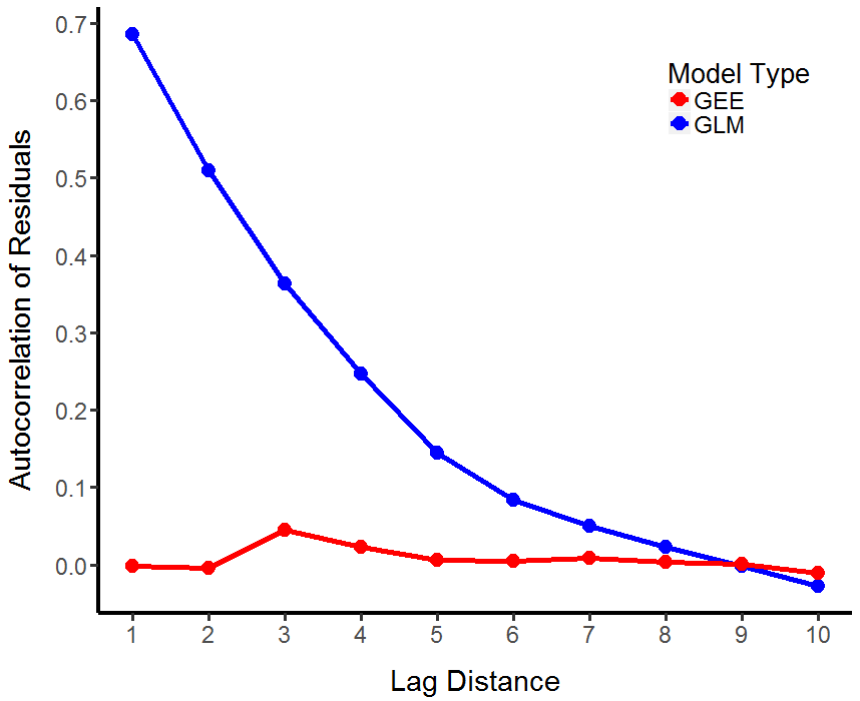
Autocorrelation of residuals from GEE and in comparison to GLM. GEE with correlation structure: fixed performed best for the musdata data set. Spatial autocorrelation is computed as Moran’s I using the *acfft *function. The figure depicts simulated occurrence data of *Mus musculus *in response to the degree of pollution and the degree of exposure (for instance, to light, noise or other hypothetical risk factors).

**Figure 2.**
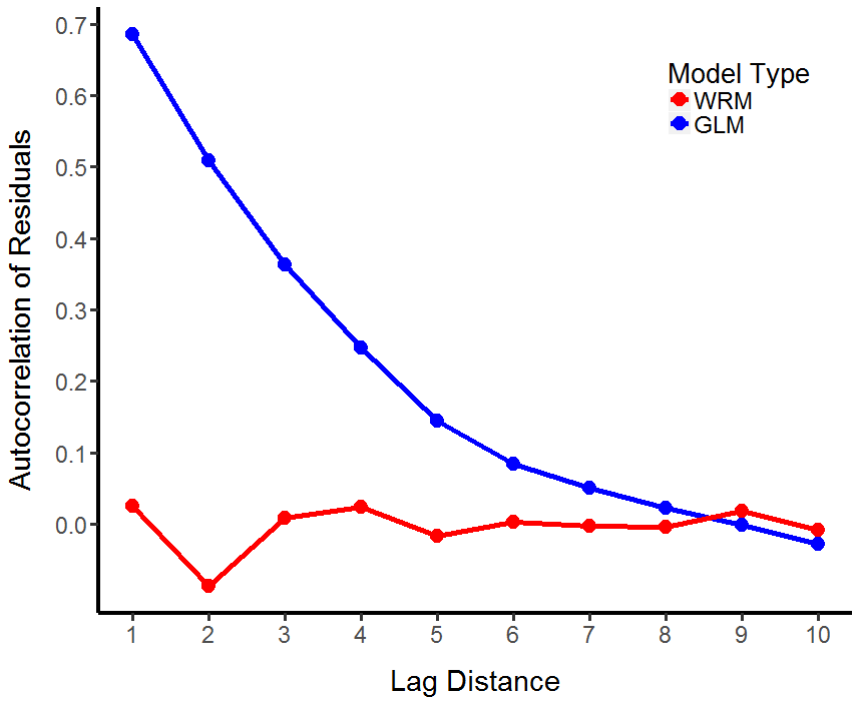
Autocorrelation of residuals from WRM in comparison to GLM. WRM with level 1 performed best for the musdata data set. Spatial autocorrelation is computed as Moran’s I using the *acfft *function.

**spind** includes methods for GEEs (*summary.GEE* and *predict.GEE*). These are useful in evaluating model fit and autocorrelation of residuals compared to a non-spatial model (in** spind**, this is a GLM with the same family as the GEE). Additionally, one can use the *plot *and *customize_plot* arguments in GEE to visually inspect the autocorrelation of the residuals from each regression and edit the plot using *ggplot2* style inputs (Fig. 1). For GEE models, the authors use normalised Pearson residuals, which are normalised in terms of correlation, to check whether and how far the autocorrelation is reduced. Note that a QIC (Quasi-information Criterion) score is reported as opposed to AIC. This is calculated based on the method described in Hardin and Hilbe (2003) (see also: Barnett et al. 2010) and is implemented using the function *qic.calc*.

One drawback to non-clustered methods for GEEs arises from the way that *R *handles matrices. Trying to fit GEEs with *corstr="fixed"* to large data sets (i.e. the number of observations is approximately *sqrt(.Machine$integer.max)*) will result in errors, as the resulting variance-covariance matrices will be too large to be handled in *R*. This is where fitting clustered models is useful, as they work with smaller, more manageable matrices. These can be specified by changing the *corstr* argument to either *"quadratic"* or *"exchangeable"*.

## Specification

### Other features specific to wavelets

The package includes other functions that may be useful in diagnosing scale-specific features.

For example, the user might want to plot the wavelet variance or covariance of each of the variables as a function of level. The *covar.plot* function allows the user to visually examine the wavelet relationships from the model. For wavelet variance and covariance computation, the wavelet family *d4* and the *wtrafo = "modwt"* were found to be mathematically more appropriate than others (Carl et al. 2016).

*covar.plot(carlina.horrida ~ aridity + land.use - 1,*

*data = carlinadata, coord = coords, wavelet = "d4",*

*wtrafo = "modwt", plot = "var")*

*covar.plot(carlina.horrida ~ aridity + land.use - 1,*

*data = carlinadata, coord = coords, wavelet = "d4",*

*wtrafo = "modwt", plot = "covar")*

**Figure 3.**
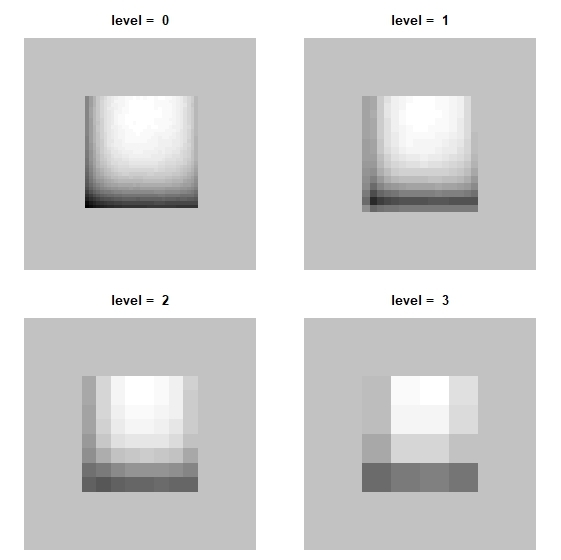
Smooth components of wavelet decompositions at different scale levels. The upscaling is performed by the upscale function for variable aridity belonging to *carlinadata* data set. The data represent a square region. (Any region is extended to the next or next but one square of 2^n^x2^n ^grid cells and is padded with predefined values, default is mean value, by the function provided. Thus the data recorded is available in a form that enables wavelet analyses.) Level=0 displays the raw, full-resolution predictor values, which are then "aggregated" by wavelets to ever coarser resolutions. Values increase from black to white. This function can be applied to any variable of interest, e.g. predictor, response or residuals.

The user may also want to view the smooth (i.e. slowly varying) components of any spatial data set at different resolutions. For this, the upscale function is offered, which allows the user to visually examine his/her data. The data sampled on a grid of squared cells and mostly sampled on adjacent cells may have any external contour. A square is used that embeds or completely encapsulates the data. Therefore, it becomes visible as a surface in a square. This square consists of 2^n ^x 2^n^ grid cells allowing a dyadic up-scaling for a number of different levels of *scale*. This means that the procedure of up-scaling related to the resolution level can be imagined as a two-dimensionally gradual enlargement of cell sizes (Carl et al. 2016). The function *upscale* offers the option to adjust padding settings so the user can see how that influences the results. The default is mean values of the input vector, but it can be easily switched using the *pad* argument, which works the same way as in the other WRM functions. In the example below, one of the covariates of the *carlinadata* data set (Fig. 3) is used*.* As can be seen, in this case, the external structure of the data set is a square.

*upscale(f = carlinadata$aridity, coord = coords)*

### Multi-model inference with GEEs, WRMs, and WMRRs

**spind** provides a couple of frameworks for conducting multi-model inference analyses (Burnham and Anderson 2002). The first that is introduced here is the *step.spind* function, which implements step-wise model selection. The process is loosely based on *MASS::stepAIC* and *stats::step*, but is specific to classes *GEE* and *WRM*. For GEEs, *step.spind* uses models with the lowest QIC scores to determine what the next step will be. For WRMs, one has the option of using AIC or AICc (AIC corrected for small sample sizes) using the logical *AICc *argument.

Currently, the function only supports backwards model selection. In other words, one has to start with a full model (i.e. all of the variables in your model formula) and they are removed in a stepwise fashion. It is intended to add forward model selection methods shortly. Additionally, step.spind is written to always respect the hierarchy of variables in the model and the user cannot override this currently. For example, *step.spind* would not remove a main effect if the variable was still present as an interaction or polynomial, e.g. removing *race* while retaining *I(race^2)*.

An example of *step.spind *using a GEE on the *birthwt* data set is shown in the **MASS** package below. The data in *birthwt* are not actually spatially explicit; a grid structure is imposed on them artificially. However, it is hoped that in using this data set, it will be demonstrated how this function can work with many types of data.

*library(MASS)*

*data(birthwt)*

*# impose an artificial (not fully appropriate) grid structure*

*x <- rep(1:14,14)*

*y <- as.integer(gl(14,14))*

*coords <- cbind(x[-(190:196)],y[-(190:196)])*

*formula <- formula(low ~ age + lwt + race + smoke + ftv + bwt + I(race^2))*

*mgee <- GEE(formula = formula, family="gaussian", data=birthwt,*

*coord=coords, corstr="fixed",scale.fix=TRUE)*

*mwrm <- WRM(formula = formula, family="gaussian", data=birthwt,*

*coord=coords, level=1)*

*ssgee <- step.spind(object = mgee,data = birthwt)*

*sswrm <- step.spind(object = mwrm, data = birthwt, AICc=TRUE, trace = FALSE)*

*best.mgee <- GEE(formula = ssgee$model, family = "gaussian", data=birthwt,*

*coord=coords, corstr="fixed",scale.fix=TRUE)*

*best.wrm <- WRM(formula = sswrm$model, family="gaussian", data=birthwt,*

*coord=coords, level = 1)*

*summary(best.mgee,printAutoCorPars=FALSE)*

*summary(best.wrm)*

Additionally, multimodel inference tools are offered for GEEs, WRMs and WMRRs which are loosely based on the **MuMIn** package. These are implemented in *mmiWMRR* and *mmiGEE*. They enable the user to examine the effect that the grid resolution and variable selection have on the resulting regressions and then to select the appropriate model for subsequent analyses. Note that *mmiWMRR* has two more arguments than *mmiGEE*. Moreover, settings will be changed in *WRM* and *scaleWMRR *for illustrative purposes. For two-dimensional wavelet models in geographical applications, the wavelet family d4 was found to be mathematically appropriate as well (Carl et al. 2016). The *padzone* has been increased from 1 to 1.1 to account for embedding in a variety of geographical areas.

*# Example for WRMs*

*data(carlinadata)*

*coords <- carlinadata[,4:5]*

*wrm <- WRM(carlina.horrida ~ aridity + land.use, family = "poisson",*

*data = carlinadata, coord = coords,level=1,wavelet="d4",pad=list(padzone=1.1))*

*ms1 <- scaleWMRR(carlina.horrida ~ aridity + land.use, family = "poisson",*

*data = carlinadata, coord = coords,scale=1,wavelet='d4', pad=list(padzone=1.1),trace=TRUE)*

*mmi <- mmiWMRR(object = wrm, data=carlinadata, scale=1, detail=TRUE,trace=TRUE)*

*# Example for GEEs*

*library(MASS)*

*data(birthwt)*

*# impose an artificial (not fully appropriate) grid structure*

*x <- rep(1:14,14)*

*y <- as.integer(gl(14,14))*

*coords <- cbind(x[-(190:196)],y[-(190:196)])*

*formula <- formula(low ~ race + smoke + bwt)*

*mgee <- GEE(formula = formula, family = "gaussian", data = birthwt,*

*coord=coords, corstr="fixed", scale.fix=TRUE)*

*mmi <- mmiGEE(object = mgee, data = birthwt)*

Finally, one further tool is offered for model selection specific to WMRRs. *rvi.plot* uses *mmiWMRR* and creates a plot of the relative importance for each explanatory variable as a function of the resolution of the grid (in other words, as a function of the *scale* argument in *mmiWMRR*). It will also print the resulting model selection tables to the console. The output can help the user in choosing the most appropriate variables for subsequent analyses.

*data(carlinadata)*

*coords <- carlinadata[,4:5]*

*rvi.plot(carlina.horrida ~ aridity + land.use, family = "poisson",*

*data=carlinadata,coord=coords,maxlevel=4,detail=TRUE,wavelet="d4",trace=TRUE)*

### Goodness of fit and model performance

Using an appropriate accuracy measure is essential for assessing prediction quality in modelling spatially explicit data. Goodness of fit measures such as Cohen's kappa coefficient, receiver operating characteristic (ROC), the area under the ROC curve (AUC) and maximum true skill statistic (TSS) are widely used to assess prediction errors in presence/absence models. There are some problems, especially related to prevalence, with more traditional measures such as AUC and hence TSS is recommended nowadays (e.g. Lobo et al. 2008, Jiménez-Valverde et al. 2008, Jiménez-Valverde 2014). Despite these warnings, these measures have been implemented to facilitate comparisons with older papers and their results. In a spatial context, however, these measures can be misleading. The reason is that a false prediction has the quality of being false regardless of its distance to an appropriate actual (observed) value and thus true prediction. One can indeed ask the question: Is a false prediction of presence in close proximity to a true (observed) presence better than a false presence far away from an observed presence (Fielding et al. 2002)? This might be the case, especially when sampling at nearby locations leads to sample values that are not statistically independent from each other. This phenomenon of statistical dependence caused by spatial proximity should be considered as relevant. For sampling on raster maps, in particular, the assignment of values to cells is arbitrary to the extent that the specification of cell size and grid orientation is arbitrary as well. Using a refined weighting pattern in a 4x4 contingency table, the above-mentioned measures were modified and improved to spatially corrected versions, which are sensitive to the spatial arrangement of predictions (Carl and Kühn 2017). This approach is even recommended when using methods to account for spatial autocorrelation in the residuals (such as GEE or WRM), because it takes account of autocorrelation in the dependent variable when comparing different model outputs.

In **spind*, ***these metrics are categorised according to whether or not their outputs are dependent on the chosen threshold. *th.dep* (threshold dependent) and *th.indep* (threshold independent) are designed to work on any number of model types; all that is needed is a set of actual values, predictions and their associated coordinates. The hook data set is used to see how these work.

*data(hook)*

*df <- hook[,1:2]*

*coords <- hook[,3:4]*

*# Threshold dependent metrics*

*th.dep.indices <- th.dep(data=df,coord=coords,spatial=TRUE)*

*# Confusion Matrix*

*th.dep.indices$cm*

*# Kappa statistic*

*th.dep.indices$kappa*

*# Threshold independent metrics*

*th.indep.indices <-th.indep(data=df,coord=coords,spatial=TRUE,plot.ROC=TRUE)*

*# AUC*

*th.indep.indices$AUC*

*# TSS*

*th.indep.indices$TSS*

### Spatial Autocorrelation

Finally, the authors would like to mention that, in many of these analyses, it is necessary to calculate spatial autocorrelation using Moran's I function (Carl and Kühn 2007). While there are many versions of this analysis in other packages, this package provides improved efficiency through the use of Fast Fourier Transforms. This is implemented in the function *acfft (AutoCorrelationFastFourierTransform)*. For illustration, a quick example computing a GLM for the *musdata* data set and calculating spatial autocorrelation of model residuals is provided.

*coords <- musdata[,4:5]*

*mglm <- glm(musculus ~ pollution + exposure, family = "poisson", data = musdata)*

*ac <- acfft(coord = coords, f = resid(mglm, type = "pearson"), lim1 = 0, lim2 = 1, dmax = 10)*

*ac*

Note that you can adjust the number of distance bins to examine in *acfft* using the *dmax* argument. The default is 10. Moreover, the user can choose the limits for the first bin (*lim1,lim2*). Its difference acts as an increment for all the others.

## Developer Notes

First, while there are many packages and functions available accounting for spatial autocorrelation in linear modeling or generalised linear modeling like frameworks, this is the only one that offers the user-optimised regression methods: spatial GEE, spatial WRM and scale-specific WMRR in conjunction with extended methods, step-wise model selection and multi-model inference analysis. Advantages are its simplicity to use as well as it computationally efficiency.

Second, to the best of the authors' knowledge, no other package or commercially available software offers the possibility to assess the accuracy of model predictions taking spatial dependence into account and being comparable to classical measures of model accuracy.

It is therefore believed that **spind** is an extremely useful tool in spatial analyses of lattice data, whether in biology, ecology, economics, geology, climatology or any other discipline with spatially structured data.

## Web location (URIs) and repository

The package, together with documentation, is available on CRAN: https://CRAN.R-project.org/package=spind. The development version can be found on GitHub: https://github.com/levisc8/spind.

## Usage rights

It is open-source software (published under the GPL public licence, ver. 3).
